# Postnatal Corticosteroids for Prevention and Treatment of Chronic Lung Disease in the Preterm Newborn

**DOI:** 10.1155/2012/315642

**Published:** 2011-10-04

**Authors:** Sachin Gupta, Kaninghat Prasanth, Chung-Ming Chen, Tsu F. Yeh

**Affiliations:** ^1^Department of Pediatrics, John Stroger Jr. Hospital of Cook County, Chicago, IL 60612, USA; ^2^Department of Pediatrics, Taipei Medical University Hospital, Taipei 110, Taiwan; ^3^Department of Pediatrics, School of Medicine, College of Medicine, Taipei Medical University, Taipei 110, Taiwan; ^4^Department of Pediatrics, China Medical University, Taichung 40402, Taiwan; ^5^Department of Pediatrics, Shuang Ho Hospital, Taipei Medical University, Taipei 110, Taiwan; ^6^Maternal Child Health Research Center, Taipei Medical University, Taipei 110, Taiwan

## Abstract

Despite significant progress in the treatment of preterm neonates, bronchopulmonary dysplasia (BPD) continues to be a major cause of neonatal morbidity. Affected infants suffered from long-term pulmonary and nonpulmonary sequel. The pulmonary sequels include reactive airway disease and asthma during childhood and adolescence. Nonpulmonary sequels include poor coordination and muscle tone, difficulty in walking, vision and hearing problems, delayed cognitive development, and poor academic achievement. As inflammation seems to be a primary mediator of injury in pathogenesis of BPD, role of steroids as antiinflammatory agent has been extensively studied and proven to be efficacious in management. However, evidence is insufficient to make a recommendation regarding other glucocorticoid doses and preparations. Numerous studies have been performed to investigate the effects of steroid. The purpose of this paper is to evaluate these studies in order to elucidate the beneficial and harmful effects of steroid on the prevention and treatment of BPD.

## 1. Introduction

Despite significant progress in the treatment of preterm neonates, bronchopulmonary dysplasia (BPD) continues to be a major cause of neonatal morbidity. At earlier times, it was considered to be primarily iatrogenic in etiology as a consequence of crude ventilator techniques. In current time with advanced and sophisticated ventilator techniques, BPD continued to be a major sequel of neonatal respiratory distress syndrome (RDS), primarily because of better survival of extreme premature babies with other factors including ventilator-induced lung injury, exposure to oxygen, and inflammation. New bronchopulmonary dysplasia (new BPD) is characterized, in part, by arrested alveolar and vascular development of the immature lung [1]. Affected infants suffer from long-term pulmonary and nonpulmonary sequel. The pulmonary sequels include reactive airway disease and asthma during childhood and adolescence [2, 3]. Nonpulmonary long-term sequels include poor coordination and muscle tone, difficulty in walking, vision and hearing problems, delayed cognitive development, and poor academic achievement [4]. 

The proposed etiology of new BPD is the initiation of inflammatory mediators that cause impairment of alveolarization and vasculogenesis [5]. The lacking anti-inflammatory mediators in the preterm neonate may be inundated easily by the proinflammatory cascade. A difference in the release of pro- and anti-inflammatory cytokines, occurring as a result of intrauterine/postnatal infection (sepsis), ventilator trauma, oxidants, pulmonary edema, or sepsis, damages the immature lung. 

As inflammation seems to be primary mediator of injury in pathogenesis of BPD, role of steroids as anti-inflammatory agent has been extensively studied and proven to be efficacious in management. But studies in last one and half decade have seriously questioned the routine use of steroids especially high-dose dexamethasone due to its long-term effect on neurodevelopment. In 2010, the American Academy of Pediatrics (AAP) revised policy statement regarding the use of postnatal corticosteroids for prevention or treatment of chronic lung disease in preterm infants, concluded that high-dose dexamethasone (0.5 mg/kg/day) does not seem to confer additional therapeutic benefit over lower doses, and is not recommended. Evidence is insufficient to make a recommendation regarding other glucocorticoid doses and preparations. The clinician must use clinical judgment when attempting to balance the potential adverse effects of glucocorticoid treatment with those of BPD. Postnatal use of dexamethasone for BPD has decreased since the publication of the AAP statement in 2002; however, the incidence of BPD has not decreased [6]. Instead, several reports have suggested that the incidence or severity of BPD may have increased. Despite AAP statement to limit the use of systemic dexamethasone especially high dose, seems reasonable considering it has proven adverse effect on neurodevelopment. But that cannot negate the fact that steroids do have beneficial effects on pulmonary physiology, and currently we do not have any other anti-inflammatory of similar efficacy. If we can limit the systemic side effects of steroid in some way and can utilize its local anti-inflammatory effect on lung, it can be a very useful drug in management of new BPD. 

Various mechanisms have been described for beneficial effect of steroids on lung mechanics in infants with BPD. Various steroids of different potency have been studied at various timings; in different dosing regimens; for different duration; in different forms (including intravenous, inhalational, intratracheal, and recently intratracheal with surfactant as a vehicle). Amongst systemically used steroids, dexamethasone comes as the most potent and most studied one. It has been studied in early (<7 days), moderately early (7–14 days) and late/delayed (>14 days), postnatal periods and dosing ranging from 0.1 mg/kg/day to 0.5 mg/kg/day and duration ranging from 3 days to 42 days. Hydrocortisone comes second. Beclomethasone is the most commonly used inhalational steroid for BPD. Recently, budesonide has been tried as intratracheal instillation with or without surfactant as a vehicle and shown to reduce inflammatory marker in tracheal aspirates in initial clinical trials.

## 2. Possible Mechanisms of Action of Glucocorticoids

As the pathogenesis of BPD is multifactorial, so are the mechanisms to respond to steroid therapy. Since inflammation seems to play a critical role in the evolution of BPD, benefit seen with glucocorticoids most likely mediates through its anti-inflammatory effect. 

The primary anti-inflammatory effect of glucocorticoids is mediated by annexin-1 synthesis. Annexin-1 suppresses phospholipase A2 expression, thereby blocking eicosanoids (i.e., prostaglandins, thromboxanes, prostacyclins, and leukotrienes) and the subsequent leukocyte inflammatory events including adhesion and migration. Thus, glucocorticoids inhibit two main products of inflammation prostaglandins and leukotrienes. In addition, glucocorticoids also suppress both cyclooxygenase I and II similar to NSAID, potentiating the anti-inflammatory effect [7].

Lung inflammation is downregulated by dexamethasone therapy. Groneck et al. evaluated the tracheobronchial aspirate from preterm infants at high risk of BPD. The number of neutrophils and concentrations of leukotriene B4, interleukin-1, elastase-*α*1-protease inhibitor, and albumin were decreased after dexamethasone treatment [8]. It indicates that dexamethasone affects the release of inflammatory mediators and neutrophils influx into the airways of preterm infants who require mechanical ventilation and decreases the microvascular permeability. Pulmonary edema is the hallmark of BPD; dexamethasone has been shown to reduce the pulmonary edema in infants with BPD.

Glucocorticoids block the release of arachidonic acids and its subsequent conversion to eicosanoids. The decreased incidence of patent ductus arteriosus (PDA) after prenatal or postnatal steroid therapy is likely due to the influence of the corticosteroid effect on the responsiveness of ductal tissue to prostaglandins. Prostaglandin has an important role in maintaining the integrity of gastrointestinal mucosa. The use of steroids may increase the risk of gastrointestinal perforation. Other mechanisms such as modulating the transcription and posttranscriptional regulation of surfactant component, stimulation of antioxidant production, and enhancement of adrenergic activities may also be responsible for the acute and rapid improvement of pulmonary function [9]. Unfortunately, some of these mechanisms are also involved in physiologic signaling other than inflammatory signaling; the therapeutic effects of glucocorticoids in inflammation are often accompanied by clinically significant side effects. Glucocorticoid receptors are present virtually in all cells. Prolonged or high-dose glucocorticoids therapy causes multiple systemic side effects. There is a consensus that the desired anti-inflammatory effects of glucocorticoids are mainly mediated via repression of gene transcription. In contrast, the underlying molecular mechanisms for glucocorticoids-mediated side effects are complex and partly understood.

## 3. Postnatal Corticosteroid Therapy in Preterm Infants

### 3.1. Choice of Glucocorticoids

Dexamethasone is a potent, long-acting steroid with exclusive glucocorticoid effect. When compared to hydrocortisone, dexamethasone is 25–50 times more potent. The half-life is 36–54 hours. Dexamethasone has been extensively studied in neonatal medicine and has shown to improve pulmonary function, facilitate extubation, and decrease the incidence of BPD [10–15]. However, many associated adverse side effects prevent the routine use of dexamethasone. The short-term side effects include hyperglycemia, hypertension, hypertrophic cardiomyopathy, gastrointestinal bleeding, and perforation. The risk of gastrointestinal perforation increases with concomitant indomethacin treatment [16]. There is also a concern with the chronic suppression of the hypothalamic-pituitary-adrenal axis [17, 18] and long-term neurodevelopmental delay [19, 20].

On the other hand, hydrocortisone has almost equal glucocorticoid and mineralocorticoid action, and the half-life is only 8 hours. Sick premature infants have relative adrenal insufficiency during acute illness because of developmental immaturity of the hypothalamic-pituitary-adrenal axis suggesting that an early physiological replacement of cortisol may be needed [21–24]. However, large doses above physiologic levels to achieve the anti-inflammatory action may cause significant mineralocorticoid side effects. Early use of hydrocortisone (<48 hours) was shown to decrease the risk of PDA but increased survival only in infants exposed to maternal chorioamnionitis or who had low cortisol values [22, 23].

Another steroid betamethasone, a stereoisomer of dexamethasone, differs only in the orientation of the methyl group at position 16. However, this structural difference could be responsible for marked differences in nongenomic effects. Previous antenatal steroid studies have demonstrated that both drugs have the same effects in reducing the risk of intraventricular hemorrhage, but betamethasone has been shown to be more effective than dexamethasone in reducing the risk of neonatal death and cystic periventricular leukomalacia among very premature infants [24, 32]. The study of betamethasone in postnatal use is limited. A recent study has shown that betamethasone is as effective as dexamethasone in improving pulmonary function, but with fewer adverse effects, such as poor weight gain and hyperglycemia [33].

Inhaled glucocorticoids have been used in neonates without concomitant systemic side effects. They have been successfully used for years in asthmatic patients, but their effects on mechanical ventilated preterm infants are less impressive. The delivery of inhaled glucocorticoids in preterm infants is technically difficult, and its effectiveness has been shown to be limited. Similarly, direct intratracheal instillation of glucocorticoids alone has also not been shown to be effective. A topical glucocorticoid aerosol (budesonide, fluticasone, or beclomethasone) is administered by metered dose inhaler and spacer directly to the endotracheal tube of intubated infants. In an animal model, delivery of beclomethasone to the lungs of an intubated neonate was only 1-2% of the original aerosolized drug [34]. The inhaled steroid did not decrease the incidence of BPD but improved blood gas, chest X-ray score, and a decrease in the use of systemic steroids [35–38].

A recent study from Yeh et al. suggested that intratracheal instillation of budesonide, a strong local glucocorticoids, using surfactant as vehicle may effectively deliver the medication to the lung and may decrease the incidence of BPD [39].

### 3.2. Timing of Postnatal Steroid Use

The potential mechanism of glucocorticoids in premature infants with RDS is not exactly known. Most of the clinical trials only evaluated clinical responses and did not study mechanisms explaining the beneficial effects. Based on the pathologic and physiologic studies, it seems that steroid therapy given at different times may mediate physiologic effect via different mechanisms. Premature infants may develop lung injury shortly after birth and during the first 1-2 weeks after exposure to infection, oxygen, or positive pressure ventilation. Therefore, steroid should be given shortly after birth or during the first few weeks to prevent BPD via its anti-inflammatory action. On the other hand, steroid therapy given at 3–6 weeks of life may derive its benefits from the modulation of lung repair. Alternately, steroids given at any age may be effective in infants with BPD by blunting hyperreactivity and inflammation.

### 3.3. Dosage and Duration of Corticosteroids

Most recent studies used a dose of dexamethasone 0.1–0.5 mg/kg/day, equivalent to 10 to 20 times of endogenous corticosteroid levels, in durations ranging from 3 to 42 days. The high dosage and long duration of treatment might be responsible for the delay of brain growth and subsequent poor neurodevelopmental outcomes. A lower dose and shorter duration of dexamethasone may be beneficial and without significant side effects. However, the proper dosage and duration of treatment has not been well defined.

Compare to dexamethasone, the dosage of hydrocortisone used in the trials aimed to prevent BPD was smaller, ranging from 1-2 mg/kg/day, which is equivalent to 1 to 2 times the physiological level. Unfortunately, the low-dose replacement showed no reduction of BPD.

## 4. Current Evidence of Steroid Use: AAP Revised Policy, 2010

### 4.1. Dexamethasone

Current evidence suggests that dexamethasone may decrease mortality rates, facilitate extubation, and generally decrease the incidence of BPD but that it carries a significant risk for short- and long-term adverse effects, especially impairment of growth and neurodevelopment [6, 53–56].

1. Cochrane database systemic review concluded that the benefits of dexamethasone therapy in the first week of life may not outweigh its many adverse effects [57]. In contrast, it concludes that treatment after the first postnatal week may reduce mortality rates without increasing adverse long-term neurodevelopmental outcomes although long-term follow-up data remain limited [58].
2. Two other systemic meta-analyses have been done recently. In the first review, a risk-weighted meta-analysis, the authors emphasized the importance of the a priori risk of death or BPD in different study populations [59]. In this analysis, the incidence of death or cerebral palsy (CP) was increased among dexamethasone-treated infants compared with placebo-treated infants in studies that enrolled patients at low risk (<35%) of BPD. In contrast, dexamethasone treatment decreased the risk of death or CP when infants at high risk of BPD (≥65%) were studied [59]. Thus, for infants at the highest risk of BPD, the beneficial effect of dexamethasone in reducing lung disease seemed to outweigh its adverse effect of increasing the risk of CP. In the second meta-analysis, the authors compared outcomes for trials with different cumulative doses of dexamethasone and concluded that a higher cumulative dose improved rates of survival without BPD and did not increase adverse long-term effects [60].
3. Small individual randomized controlled trials (RCTs) that directly compared high-versus low-dexamethasone doses, variably defined, have revealed no differences in efficacy (Table 1) [25–27]. These studies have generally been small and heterogeneous, which makes them difficult to compare.
4. Three RCTs have compared dexamethasone to placebo (Table 1); 1 was small and the other 2 were stopped early and are, therefore, underpowered [28–30]. One trial compared an early, short course of dexamethasone to placebo and revealed no significant difference in mortality or BPD rates [29]. The other 2 trials evaluated the efficacy of a later, lower-dose course of dexamethasone for facilitating extubation, and the authors reported that significantly more dexamethasone-treated infants were successfully extubated during the treatment period [28, 30]. Similar results were reported from an additional study that compared systemic dexamethasone to inhaled beclomethasone for extubation; significantly more dexamethasone-treated infants were successfully extubated within 7 days (Table 1) [31]. These extubation trials were not powered to evaluate the effect of the treatment on rates of survival without BPD
5. Many short-term adverse effects of dexamethasone therapy have been described; however, the main reason for the decline in its use is an adverse effect on neurodevelopment, particularly higher rates of CP. Eleven RCTs have been done to evaluate long term neurodevelopmental outcome (Table 2) [25, 40–48]. The heterogeneity of these reports makes it problematic to combine them meaningfully. Some studies did not reveal adverse effects on neurodevelopmental outcomes at various ages, whereas others did. Most of the studies were small, which reduced their ability to either prove or disprove causation. Two RCTs that used low doses of dexamethasone revealed no significant increase in CP or other neurodevelopmental impairments when compared with placebo. Because only a total of 96 dexamethasone-treated infants were evaluated in these studies, the results must be interpreted with caution [41, 42].
6. Cohort studies of dexamethasone have revealed an association of its use with impaired neurodevelopmental outcomes [48, 61]; however, such an association cannot be construed as definitive evidence of harm. A clinician's decision to use a therapy incorporates numerous undocumented factors and varies from one clinician to the next, which may seriously confound the interpretation of such studies. Patients who receive dexamethasone for BPD are likely to be perceived as having more severe respiratory disease than infants who are not treated; such infants may have worse overall outcomes regardless of dexamethasone therapy.
7. Authors of small series have also reported that infants treated with dexamethasone have more abnormalities on MRI than those not treated; again, causation cannot be attributed in the absence of an RCT [61, 62]. Two previously reported RCTs revealed more cranial ultrasound abnormalities in dexamethasone-treated infants compared with those treated with placebo, but the patient numbers were quite small [63, 64].

In summary, high daily doses of dexamethasone have been linked frequently to adverse neurodevelopmental outcomes, and this therapy is discouraged. Because an increase in adverse neurodevelopmental outcomes in treatment studies that used low doses of dexamethasone has not been reported, further studies of low-dose dexamethasone to facilitate extubation are warranted.

### 4.2. Hydrocortisone

1. Four RCTs designed to evaluate the ability of early hydrocortisone therapy to improve rates of survival without BPD have been done in recent times (Table 3) [49–52]. These studies were based on the premise that extremely preterm infants may have immature adrenal gland function, predisposing them to a relative adrenal insufficiency and inadequate anti-inflammatory capability during the first several weeks of life [21, 65–68]. In contrast to the heterogeneous nature of previous dexamethasone trials, these studies were similar in design, time of initiation, duration, and dose. The direction of effect favored the hydrocortisone-treated infants in all 4 studies, and a significant increase in rate of survival without BPD in the hydrocortisone-treated infants was reported for 2 of the studies. The largest trial (*n* = 360) did not reveal a significant benefit of hydrocortisone treatment in the overall study group; however, for infants exposed to prenatal inflammation (*n* = 149), identified before the trial as a specific group for analysis, hydrocortisone treatment resulted in a significant decrease in mortality rate and an increase in rate of survival without BPD [50]. Patient enrollment was halted early in 3 of these 4 studies because of a significant increase in spontaneous gastrointestinal perforation discovered in the largest trial [50], a complication also observed with early dexamethasone [68, 69]. The perforations may have resulted from an interaction between high endogenous cortisol concentrations and indomethacin therapy in the first 48 hours; however, because administration of indomethacin was not randomized, this hypothesis remains to be tested.
2. Neurodevelopmental outcomes at 18 to 22 months' corrected age have been published for 3 of these trials, and no adverse effects of hydrocortisone treatment were found [70, 71]. In the largest multicenter trial, the incidence of death or major neurodevelopmental impairment (52% (hydrocortisone-treated) versus 56% (placebo)), major neurodevelopmental impairment alone (39% versus 44%), and CP (16% versus 18%) was similar [70]. The only significant findings favored the hydrocortisone-treated group and included a decreased incidence of a Bayley Scales of Infant Development (2nd edition) Mental Developmental Index (MDI) 2 SDs below the mean (MDI < 70, 27% versus 37%; odds ratio: 0.47 (95% confidence interval: 0.25–0.87)) and a higher incidence of awareness of object permanence (an early test of working memory and prefrontal executive function) (89% versus 79%; odds ratio: 2.19 (95% confidence interval: 1.06–4.52)).
3. Hydrocortisone therapy given to facilitate extubation has been studied in cohort studies. In the first reported study, 25 infants treated with hydrocortisone at 1 hospital (5 mg/kg per day, tapered over 3 weeks) were compared with 25 untreated infants at the same hospital and additionally with a cohort of 23 infants treated with dexamethasone (0.5 mg/kg per day, tapered over 3 weeks) at a separate hospital [72]. The investigators found that hydrocortisone was as effective as dexamethasone in weaning infants from the ventilator and in decreasing supplemental oxygen therapy, with fewer short-term adverse effects. Followup of these children at school age revealed no differences in neurodevelopmental outcomes between hydrocortisone-treated infants and their comparison group, whereas dexamethasone-treated infants more often had an abnormal neurologic examination and less favorable school performance than their comparison cohort [72–75]. Subsequently, several large cohort studies from the same institution reported that although hydrocortisone-treated children were younger, smaller, and sicker than their untreated comparison groups, there were no adverse effects of hydrocortisone treatment on IQ, visual motor integration, memory testing, CP, or findings on MRI [74–76]. Investigators from this institution have also reported that neonatal dexamethasone but not hydrocortisone therapy resulted in long-lasting changes in hypothalamic-pituitary-adrenal axis and T-cell function [77].

### 4.3. Differences between Dexamethasone and Hydrocortisone

As discussed before, many RCTs have shown adverse neurodevelopmental outcomes after postnatal dexamethasone treatment for BPD, but neither multicenter RCTs nor cohort studies have revealed adverse effects on functional or structural neurologic outcomes after neonatal hydrocortisone therapy. Possible reasons could be as follows.

1. Dissimilar effective glucocorticoid dose-neonatal animal studies have consistently revealed adverse effects on brain growth after high doses of glucocorticoid [78, 79], and results of evaluation of 22 patients who received high-dose hydrocortisone in a study from the early 1970s were suggestive of harm [80, 81]. High-dose dexamethasone (0.5 mg/kg per day) is equivalent to at least 15 to 20 mg/kg per day of hydrocortisone [82], far higher than the doses of hydrocortisone given in the recent studies described previously. Low-dose dexamethasone (0.1–0.15 mg/kg per day) may be equivalent to 3 to 6 mg/kg per day of hydrocortisone; however, because of its much longer biological half-life, it could have a much higher relative potency [83]. Lowering the dose of dexamethasone may, therefore, decrease its adverse effects, as is suggested by the 2 studies of outcome after lower-dose dexamethasone therapy [41, 42].
2. There are dissimilar effects of these agents on the hippocampus, an area of the brain critical to learning, memory, and spatial processing [84, 85]. The hippocampus contains a high density of both mineralocorticoid and glucocorticoid receptors [86, 87]. Hydrocortisone, which is identical to native cortisol, can bind to both classes of receptors. In contrast, dexamethasone binds only to glucocorticoid receptors, which, in animal models, has been shown to result in degeneration and necrosis of hippocampal neurons [88, 89]. This effect of dexamethasone is blocked by simultaneous administration of corticosterone (the cortisol equivalent in the rat) [88]. In humans, neonatal treatment with dexamethasone, but not hydrocortisone, has been shown to alter hippocampal synaptic plasticity and associative memory formation in later life [90]. Dexamethasone exposure has also been linked to decreased hippocampal volume in 1 cohort study [91, 92], but cohort studies of infants treated with hydrocortisone have revealed no decrease in hippocampal volume [74], no adverse effect on hippocampal metabolism, and no adverse effect on memory at school age [76] when compared with a larger, more mature group of nontreated infants.

Whatever the underlying explanation(s) for the observed differences in short- and long-term outcomes may be, further RCTs are needed to answer the many remaining questions, including whether lower doses of dexamethasone can avoid previously observed adverse effects, whether hydrocortisone is efficacious for extubation, whether specific groups of infants may derive particular benefit from hydrocortisone therapy, and whether the incidence of spontaneous gastrointestinal perforation during early glucocorticoid administration can be decreased by avoiding concomitant indomethacin or ibuprofen therapy and/or by monitoring cortisol concentrations.

### 4.4. Other Glucocorticoids (Systemic)

No available evidence support use of other systemic glucocorticoids, such as prednisone or methylprednisolone, to treat or prevent BPD.

### 4.5. Inhaled Glucocorticoids

Although some tertiary care Neonatal ICUs routinely use inhaled beclomethasone for BPD babies, no available evidence support the efficacy of inhaled glucocorticoids to prevent or decrease the severity of BPD. Recent Cochrane database systemic review concluded “there is no evidence that inhaling steroids prevent chronic lung disease or the number of days the baby needed breathing support and additional oxygen” [93, 94]. 

Beclomethasone and flunisolide have been studied by nebulization in view of decreasing need for systemic steroid and side effects. The early postnatal administration of inhaled steroid to prevent BPD was studied in a large randomized, multicenter trial [34]. In this study, 253 infants with a gestation age of <33 weeks, a birth weight of <1250 g, and who were mechanically ventilated at 3 to 14 days of age were randomly assigned to inhaled beclomethasone or a placebo for four weeks. The need for supplemental oxygen was similar in the beclomethasone and placebo groups at 28 days of life and 36 weeks postmenstrual age. In this study, beclomethasone therapy did not prevent BPD; however, it significantly reduced the use of systemic glucocorticoid therapy and mechanical ventilation at 28 days of age. In a small study, fluticasone propionate inhalation was given for 3 weeks to premature infants (less than 32 weeks) with moderate BPD (required fraction of inspired oxygen >0.25 or mechanical ventilation) at 28–60 days. There was no difference between infants treated with inhaled fluticasone versus placebo in the duration of oxygen therapy or ventilatory support [32].

### 4.6. Direct Intratracheal Instillation (IT) of Steroid with or without Surfactant as a Vehicle

Aerosolized drugs may be ineffective in preterm infants as very little drug is delivered to the lung, thereby limiting its effects. Novel idea of using surfactant as a vehicle to administer budesonide has been under study. A recent study by Halliday et al. demonstrated that intratracheal instillation of budesonide using surfactant as a vehicle significantly decreased the combined outcome of death and CLD without apparent immediate and long-term adverse effects [56]. Budesonide is a strong topical anti-inflammatory glucocorticoid. It can be effectively delivered to the lungs and remain in the lungs for some time after intratracheal instillation. Once absorbed, it can be rapidly metabolized to metabolites of low glucocorticoid effect. However, before this regimen can be recommended, a large sample trial is needed.

## 5. Current Evidence-Based Recommendations (AAP Revised Policy Statement, 2010)

(1) High daily doses of dexamethasone (approximately 0.5 mg/kg per day) have been shown to reduce the incidence of BPD but have been associated with numerous short- and long-term adverse outcomes, including neurodevelopmental impairment, and at present, there is no basis for postulating that high daily doses confer additional therapeutic benefit over lower-dose therapy.
*Recommendation*. In the absence of randomized trial results showing improved short- and long-term outcomes, therapy with high-dose dexamethasone cannot be recommended.
(2) Low-dose dexamethasone therapy (<0.2 mg/kg per day) may facilitate extubation and may decrease the incidence of short- and long-term adverse effects observed with higher doses of dexamethasone. Additional RCTs sufficiently powered to evaluate the effects of low-dose dexamethasone therapy on rates of survival without BPD, as well as on other short- and long-term outcomes, are warranted.
*Recommendation*. There is insufficient evidence to make a recommendation regarding treatment with low-dose dexamethasone.

(3) Low-dose hydrocortisone therapy (1 mg/kg per day) given for the first 2 weeks of life may increase rates of survival without BPD, particularly for infants delivered in a setting of prenatal inflammation, without adversely affecting neurodevelopmental outcomes. Clinicians should be aware of a possible increased risk of isolated intestinal perforation associated with early concomitant treatment with inhibitors of prostaglandin synthesis. Further RCTs powered to detect effects on neurodevelopmental outcomes, aimed at targeting patients who may derive most benefit and developing treatment strategies to reduce the incidence of isolated intestinal perforation, are warranted.

*Recommendation*. Early hydrocortisone treatment may be beneficial in a specific population of patients; however, there is insufficient evidence to recommend its use for all infants at risk of BPD.

(4) Higher doses of hydrocortisone (3–6 mg/kg per day) instituted after the first week of postnatal age have not been shown to improve rates of survival without BPD in any RCT. RCTs powered to assess the effect of this therapy on short- and long-term outcomes are needed.

*Recommendation*. Existing data are insufficient to make a recommendation regarding treatment with high-dose hydrocortisone.

## 6. Summary

BPD is the disease of very low birth weight and extremely low birth weight newborns with multifactorial etiology including prematurity itself, ventilator-induced injury, oxygen, and inflammation. BPD has long-term adverse pulmonary and neurodevelopment outcome. Steroids usage for treatment of BPD also has been shown to have adverse neurodevelopmental outcome. Available data are conflicting and inconclusive; clinicians must use their own clinical judgment to balance the adverse effects of BPD with the potential adverse effects of treatments for each individual patient. Very low birth weight infants who remain on mechanical ventilation after 1 to 2 weeks of age are at very high risk of developing BPD [58]. When considering corticosteroid therapy for such an infant, clinicians might conclude that the risks of a short course of glucocorticoid therapy to mitigate BPD are warranted [59]. This individualized decision should be made in conjunction with the infant's parents.

## Figures and Tables

**Table 1:** RCTs of dexamethasone to prevent or treat BPD reported since 2001.

Study, no. of centers	*n*	Eligibility criteria (all on mechanical ventilation)	Timing	Dexamethasone dosing regimen	Outcome
McEvoy et al. [25], 1 center	62	500–1500 g BW; ≤32 wk gestation	7–21 postnatal days	5 mg/kg/day tapered over 7 days versus 0.2 mg/kg tapered over 7 days	Rate of survival without BPD 76% versus 73% (NS); no benefit to higher dose
Odd et al. [26], 1 center	33	≤1250 g BW	1–3 wk of age	0.5 mg/kg/day tapered over 42 days versus “individualize” (same dose, shorter course)	Rate of survival without BPD: 24% versus 30% (NS); no difference in 18-month outcomes
Malloy et al. [27], 1 center	16	<1501 g BW; <34 wk gestation	<28 postnatal days	0.5 mg/kg/day tapered over 7 days versus 0.08 mg/kg/day for 7 days	Rate of survival without BPD: 11% versus 38% (NS); higher dose had more adverse effects, no apparent benefit
Walther et al. [28], 1 center	36	≥600 g BW; 24–32 wk gestation	7–14 d postnatal age	0.2 mg/kg/day tapered over 14 days versus placebo	Rate of survival without BPD: 65% versus 47% (NS); extubation: 76% versus 42% (*P* < .05)
Anttila et al. [29], 6 centers	109	500–999 g BW; ≤31 wk gestation	Eligible at 4 h of age	0.25 mg/kg every 12 h × 4 doses versus placebo	Rate of survival without BPD: 58% versus 52% (NS)
Doyle et al. [30], 11 centers	70	<1000 g BW; <28 wk gestation	>1 wk postnatal age	0.25 mg/kg every 12 h × 4 doses versus placebo	Rate of survival without BPD: 14% versus 9% (NS); extubation: 60% versus 12% (odds ratio: 11.2 (95% confidence interval: 3.2–39.0))
Rozycki et al. [31], 1 center	61	650–2000 g BW	≥14 day postnatal age	0.5 mg/kg/day tapered over 42 day versus inhaled beclomethasone at 3 different doses for 7 days followed by the above-listed dexamethasone course, if still mechanically ventilated	Rate of survival without BPD: 53% versus 46% (NS); extubation by 7 d: 7 of 15 versus 6 of 46 (*P* < .01)

BW = body weight; NS = not significant.

**Table 2:** Neurodevelopmental follow-up of dexamethasone RCTs reported after 2001.

Study, planned age at followup	Followup, % (no. of infants seen)	Treatment start time	Dexamethasone dosing regimen	Primary neurodevelopmental findings
McEvoy et al. [25], 1 year	66 (39)	At 7–21 days	High versus low dose: 7-day taper from 0.5 mg/kg/day versus 0.2 mg/kg/day	MDI < 70: 24% (high) versus 17% (low) (NS); CP: 10% versus 11% (NS)
Armstrong et al. [40], 18 months chronological age	96 (64)	On day 7	42-d taper versus 3-day pulse	No difference in 18-month outcomes No disability: 34% versus 31% (NS)
Doyle et al. [41], 2 years corrected age	98 (58)	After 7 days	0.15 mg/kg/day tapered over 10 days	Death or major disability: 46% versus 43% (NS); death or CP: 23% versus 37% (NS); CP: 14% versus 22% (NS); major disability 41% versus 31% (NS)
Stark et al. [42], 18–22 months corrected age	74 (123)	On day 1	0.15 mg/kg/day tapered over 7 days	MDI < 70: 51% versus 43% (NS); PDI < 70: 30% versus 35% (NS); abnormal neurologic exam: 25% each group
Romagnoli et al. [43], 3 years	100 (30)	On day 4	0.5 mg/kg/day tapered over 1 wk	No differences in any parameter; CP: 9% versus 14% (NS)
Wilson et al. [44], 7 years	84 (127)	Before 3 days	4 groups: 0.5 mg/kg/day tapered over 12 days versus late (15 days) selective, versus inhaled early or late selective	No difference in cognitive, behavioral, CP, or combined outcomes
Yeh et al. [45], school age (mean: 8 years)	92 (146)	On day 1	0.5 mg/kg/day for 1 wk, then tapered for a total of 28 days	Treated children were shorter (*P* = .03), had smaller head circumference (*P* = .04), lower IQ scores (*P* = .008), and more significant disabilities (CP, IQ < 5th percentile, vision or hearing impairment): 39% versus 22% (*P* = .04)
O'Shea et al. [46], 4–11 years	89 (84)	On day 15–25	0.5 mg/kg/day tapered over 42 days versus placebo	Death or major NDI: 47% versus 41% (NS); major NDI alone: 36% versus 14% (*P* =.01)
Gross et al. [47], 15 years	100 (22)	On day 14	0.5 mg/kg/day tapered over 42 days versus 18-day taper versus placebo	Intact survival (IQ > 70, normal neurologic exam, regular classroom): 69% versus 25% (18-d course) versus 18% (placebo) (*P* < .05)
Jones and the Collaborative Dexamethasone Trial Follow-up Group [48], 13–17 years	95 (150)	At 2–12 wk	0.5 mg/kg/day for 7 days	No difference in moderate/severe disability (defined as IQ > 2 SDs < mean, CP, hearing or vision loss); CP: 24% versus 15% (relative risk: 1.58 [95% confidence interval: 0.81–3.07])

NDI: neurodevelopmental impairment; PDI: psychomotor developmental index; NS: not significant.

**Table 3:** RCTs of early hydrocortisone to prevent BPD.

Study, no. of centers	*n*	Population: mechanically ventilated infants	Timing	Hydrocortisone dosing regimen	Rate of survival without BPD HC versus placebo, %
Watterberg et al. [49], 2 centers	40	BW: 500–999 g	<48 h postnatal age	0.5 mg/kg every 12 h for 9 days 0.25 mg/kg every 12 h for 3 days	60 versus 35 (*P* = .04)
Watterberg et al. [50], 9 centers	360	BW: 500–999 g	<48 h postnatal age	0.5 mg/kg every 12 h for 12 days 0.25 mg/kg every 12 h for 3 days	35 versus 34 (OR: 1.20 (95% CI: 0.72–1.99))
Peltoniemi et al. [51], 3 centers	51	BW: 501–1250 g	<36 h postnatal age	2.0 mg/kg/day tapered to0.75 mg/kg/day over 10 days	64 versus 46 (OR: 1.48 (95% CI: 0.49–4.48))
Bonsante et al. [52], 2 centers	50	BW: 500–1249 g	<48 h postnatal age	0.5 mg/kg every 12 h for 9 days; 0.25 mg/kg every 12 h for 3 days	64 versus 32 (*P* < .05)
